# The Vascular Hypothesis of Dementia, including Alzheimer's Disease, and Risk Factors for VaD and AD in African Populations

**DOI:** 10.1002/alz70860_102699

**Published:** 2025-12-23

**Authors:** Raj Kalaria

**Affiliations:** ^1^ Translational and Clinical Research Institute, Newcastle University, Newcastle, United Kingdom; University of Nairobi, Nairobi, Kenya

## Abstract

Alzheimer disease (AD) is the considered the most common cause of dementia second to vascular dementia (VaD) worldwide. AD and VaD are multifactorial conditions also influenced by genetic and environmental factors that accelerate the global burden of cognitive impairment and dementia in an ever‐increasing ageing population even in the LMICs. Despite the best efforts to create the clinical biological construct of AD, post‐mortem findings in >80% of probable AD cases show variable degrees of vascular pathology. Frequencies of brain white matter changes explained by a vascular cause rise exponentially from as high as 20% in 60‐year‐olds with almost doubling every decade thereafter in western populations. While these frequencies from imaging studies are not known among LMIC populations, it is expected the burden is high. For example, estimates in the burden of small vessel disease markers in the data from the INTERSTROKE studies suggest incidence of ischaemic or lacunar strokes was 4.7‐fold greater in LMICs than in HICs. This translates to a high burden of vascular related cognitive impairment in Africa. Hypertension and diabetes mellitus are the main modifiable risk factors for the vascular disease. It is not clear in what ways other risks contribute to the vascular burden, but the harmful effects of hypertension are mediated by sustained structural changes in smaller arteries leading to arteriolosclerosis and fluctuations in cerebral perfusion. Cerebrovascular changes particularly arteriolosclerosis rather than infarction(s) appear early and amyloid plaque accretion occurs primarily before the onset of cognitive deficits, whereas neurofibrillary tangles, neuron loss, and particularly synaptic loss, parallel the progression of cognitive decline. The combination of vascular pathology and neurodegenerative changes are additive at each level of pathology lowering the threshold for development of AD dementia. Considering recent large sample studies, could AD therefore result from two different lobar processes such that vascular factors or disease affects the frontal lobe whereas neurodegenerative pathologies largely propagate from the temporal lobe even in African populations? Vascular disease remains a major contributor to morbidity and mortality, the whole burden of dementia can be reduced by vigorously tackling vascular risk factors at the individual, community and population level in Africa.

